# Association of Thyroid Function with Early/Mid-term Aorta-Related Adverse Events and Readmissions after Thoracic Endovascular Aortic Repair

**DOI:** 10.1038/s41598-017-15370-0

**Published:** 2017-11-07

**Authors:** Nan Lu, Zhuoqiao He, Tan Xu, Xin Chen, Xianfeng Chen, Xiaojing Ma, Xuerui Tan

**Affiliations:** 1 Department of Cardiology, the First Affiliated Hospital of Shantou University Medical College, Shantou, Guangdong, 515041 China; 2 Image Center, Wuhan Asia Heart Hospital, Wuhan, Hubei 430000 China

## Abstract

The prognosis of patients after thoracic endovascular aortic repair (TEVAR) is affected by several clinical characteristics. This study aimed to evaluate whether thyroid hormones predicts early (30 days) and mid-term (12 months) aorta-related adverse events (ARAE) and readmissions (ARAR) in patients after TEVAR. A total of 338 continuous patients who underwent TEVAR were included and stratified based on quartile of free thyroxine (FT4) levels examined before surgery. The relationship of FT4 levels with early or mid-term ARAE and ARAR were assessed using univariate and multiple logistic regression analysis. The incidence of ARAE and ARAR were 2.7% and 4.1% within 30 days, and 8.9% and 13.5% within 12 months, respectively. After adjusting for confounders, the lowest FT4 quartile group were noted to be at significantly greater risk than the highest FT4 quartile group in early (OR 10.105, 95% CI 1.103 to 92.615, *P* = 0.041) and mid-term (OR 5.687, 95% CI 1.708 to 18.935, *P* = 0.005) ARAR, but not significantly different in early (OR 2.097, 95% CI 0.228 to 19.307, *P* = 0.513) and mid-term (OR 0.695, 95% CI 0.207 to 2.332, *P* = 0.556) ARAE. Thus, patients with low-normal FT4 levels after TEVAR are at greater risk of ARAR, but not ARAE, in both the early and the mid-term follow-up periods.

## Introduction

Thoracic endovascular aortic repair (TEVAR) has been increasingly applied to treat a variety of aortic diseases^1,2^. Nevertheless, it is still a young technology with several unknowns, including the risk factors for the prognosis of patients after TEVAR. Thyroid hormones play important roles in the development and functioning of the circulatory system. Several studies have demonstrated that overt thyroid disorders, or even subclinical dysfunction, are associated with cardiovascular disease^3–7^. Blood pressure and heart rate, which can be affected by thyroid hormones, are associated with the prognosis of patients after TEVAR^3,4,8,9^.

It is unclear whether thyroid hormone levels are related to aortic diseases and whether thyroid hormones could provide valuable predictive information for patients after TEVAR. The aim of this study was to investigate the influence of thyroid hormones on aorta-related adverse events (ARAE) and readmissions (ARAR) during the early (30 days) and mid-term (12 months) follow-up periods after TEVAR.

## Methods

### Study population

A retrospective study was conducted on prospectively collected data of aortic disease patients undergoing TEVAR from January 2004 to December 2015 at Wuhan Asia Heart Hospital. Participants who had a medical history of aortic disease, Marfan syndrome or other connective tissue disease, cancer, renal insufficiency, and clinical hyperthyroidism or hypothyroidism were excluded. Participants who were scheduled to have or who underwent surgical procedures within 30 days of study enrolment were also excluded.

The methods in the study were in accordance with relevant guidelines and the Declaration of Helsinki. Written informed consent was obtained from all participants. All procedures were approved by the Ethics Committee of Wuhan Asia Heart Hospital and the Ethics Committee of The First Affiliated Hospital of Shantou University Medical College.

### Procedures

All subjects routinely received a contrast-enhanced computed tomography (CT) scan before a TEVAR procedure. The indications for the TEVAR procedure were aortic dissection (AD), intramural hematoma (IMH), aortic aneurysm (AA), penetrating aortic ulcer (PAU), and traumatic aortic lesions^10,11^. Successful procedures were defined as technically accurate placement of the stent graft at the intended target location without endoleak^11^.

Subjects were classified into the acute group (≤14 days), the sub-acute group (15–90 days), and the chronic group (>90 days), based on the time interval from symptom onset date to procedure date^10,11^.

### Diagnostic criteria

Concentrations of serum free triiodothyronine (FT3), free thyroxine (FT4) and thyroid-stimulating hormone (TSH) were measured on fasting, morning samples using 2 different assays. Hyperthyroidism and hypothyroidism were defined as serum TSH < 0.45 μU/L and TSH > 19.9 μU/L respectively^5^. All participants were stratified by quartiles based on of FT4 levels.

Persistent or unrelenting pain despite having received maximal medical therapy was considered as refractory pain^12^. Refractory hypertension was defined as persistent hypertension despite having received ≥3 different classes of antihypertensive therapies at best recommended or maximum tolerated doses^12,13^. Rupture or impending rupture was defined as extravasation of fresh blood outside the thoracic aorta or concomitant hemothorax documented by preoperative CT^14^.

### Follow-up

All subjects were followed up by 3 clinical cardiologists at 30 days, 3 months, 6 months, and 12 months from the completion of the TEVAR procedure. Symptoms, medications, laboratory measurements, electrocardiogram and imaging tests (ultrasonic cardiogram and contrast-enhanced CT, depending on medical criteria) were collected by electronic data capture and telephone interviews. Early (30 days) and mid-term (12 months) ARAE and ARAR were the primary outcomes of our study.

ARAE was defined as aorta-related death, progression of aortic disease, organ failure or lower limb ischemia, aortic expansion of >5 mm, or endoleak. ARAR was defined as readmissions associated with any aorta-related complications, such as progression of aortic disease, organ failure or lower limb ischemia, surgical requirement, or occurrence of similar symptoms upon aortic disease onset without other diseases identified by a comprehensive clinical examination.

### Statistical analysis

Continuous variables were presented as means ± standard deviations or medians (quartile 1 to quartile 3 [Q1–Q3]). Categorical variables were shown as counts and percentages. Analysis of variance, Kruskal-Wallis test, and Chi-square test were used to compare the difference in normal continuous variables, non-normal continuous variables and categorical variables, respectively. The relationship between thyroid hormones and 30-day or 12-month ARAE and ARAR were assessed using univariate and multiple logistic regression analysis. Candidate covariates for multivariable modelling were selected based on p-value (*P*) < 0.1 in univariate logistic regression analysis. Odds ratios (ORs) were presented with 95% confidence interval, and a two-tailed *P* < 0.05 was considered statistically significant. All data analyses were performed using the statistical software Statistical Product and Service Solution (SPSS 19.0 for Windows, Chicago, Illinois, USA).

### Data availability statement

All raw data and analysis code are available from the corresponding author on reasonable request.

## Results

### Demographics

Three hundred and thirty-eight subjects (mean age 56.5 years, 276 males, 62 females) were included: 221 patients with AD, 78 patients with IMH, 32 patients with AA, and 7 patients with PAU. There were 182 subjects in the acute phase, 85 subjects in the sub-acute phase, and 71 subjects in the chronic phase.

The clinical and operative procedure characteristics of subjects stratified by FT4 levels are shown in Table 1 and Table 2, respectively. None of the parameters were significantly different among the 4 levels. The medians (Q1–Q3, ng/dl) of the FT4 quartiles were 0.82 (0.76–0.86), 0.95 (0.92–0.98), 1.12 (1.07–1.17), and 1.35 (1.28–1.49), respectively.

**Table 1:** Population clinical characteristics stratified by FT4.

	FT4 level				All (n = 338)	*P value*
	Q1 (n = 77)	Q2 (n = 87)	Q3 (n = 92)	Q4 (n = 82)		
Male, n (%)	64(83.1)	69(79.3)	79(85.9)	64(78.0)	276(81.7)	0.527
Age (year)	56.9 ± 9.7	56.4 ± 10.4	55.6 ± 10.5	57.1 ± 10.2	56.5 ± 10.2	0.779
BMI (kg/m^2^)	24.86 ± 3.42	24.81 ± 3.05	24.49 ± 3.34	25.01 ± 3.62	24.78 ± 3.35	0.752
Tobacco abuse, n (%)						0.057
None	21(27.3)	39(44.8)	26(28.3)	28(34.1)	114(33.7)	
Current	46(59.7)	40(46.0)	50(54.3)	42(51.2)	178(52.7)	
Former	10(13.0)	8(9.2)	16(17.4)	12(14.6)	46(13.6)	
Alcohol abuse, n (%)						0.299
None	53(68.8)	65(74.7)	75(81.5)	65(79.3)	258(76.3)	
Current	22(28.6)	19(21.8)	14(15.2)	12(14.6)	67(19.8)	
Former	2(2.6)	3(3.4)	3(3.3)	5(6.1)	13(3.8)	
Hypertension, n (%)	63(81.8)	76(87.4)	84(91.3)	74(90.2)	297(87.9)	0.250
DM, n (%)	9(11.7)	8(9.2)	14(15.2)	7(8.5)	38(11.2)	0.488
CAD, n (%)	16(20.8)	22(25.3)	12(13.0)	18(22.0)	68(20.1)	0.213
Hypercholesterolemia, n (%)	20(26.0)	29(33.3)	28(30.4)	18(22.0)	95(28.1)	0.373
COPD, n (%)	7(9.1)	4(4.6)	2(2.2)	5(6.1)	18(5.3)	0.245
PAD, n (%)	39(50.6)	34(39.1)	33(35.9)	32(39.0)	138(40.8)	0.239
SAS, n (%)	1(1.3)	1(1.1)	1(1.1)	1(1.2)	4(1.2)	0.999
CVD, n (%)	18(23.4)	29(33.3)	19(20.7)	26(31.7)	92(27.2)	0.169
History of cardiac surgery, n (%)	0(0)	1(1.1)	2(2.2)	0(0)	3(0.9)	0.360
FT3 (pg/ml), median (IQR)	2.90(2.69–3.18)	2.91(2.71–3.18)	2.90(2.57–3.32)	2.93(2.62–3.25)	2.91(2.64–3.25)	0.942
TSH (uIU/ml)	2.31 ± 2.49	1.85 ± 1.56	1.85 ± 1.86	1.98 ± 1.40	1.99 ± 1.86	0.355
GFR (ml/min)	89.95 ± 19.91	89.62 ± 21.61	87.71 ± 23.65	84.42 ± 21.61	87.91 ± 21.83	0.346

BMI, body mass index; DM, diabetes mellitus; CAD, coronary artery disease; COPD, chronic obstructive pulmonary disease; PAD, peripheral arterial disease; SAS, sleep apnea syndrome; CVD, cerebrovascular diseases; IQR, interquartile range; FT3, free triiodothyronine; TSH, thyroid-stimulating hormone; GFR, glomerular filtration rate; FT4, free thyroxine.

**Table 2:** Population operative characteristics stratified by FT4.

	FT4 level				All (n = 338)	*P value*
	Q1 (n = 77)	Q2 (n = 87)	Q3 (n = 92)	Q4 (n = 82)		
Stage of aortic diseases, n (%)						0.251
Acute	44(57.1)	47(54.0)	53(57.6)	38(46.3)	182(53.8)	
Sub-acute	16(20.8)	24(27.6)	25(27.2)	20(24.4)	85(25.1)	
Chronic	17(22.1)	16(18.4)	14(15.2)	24(29.3)	71(21.0)	
Pathology of aortic diseases, n (%)						0.696
AD	56(72.7)	55(63.2)	59(64.1)	51(62.2)	221(65.4)	
IMH	10(13.0)	20(23.0)	24(6.1)	24(29.3)	78(23.1)	
AA	9(11.7)	7(8.0)	9(9.8)	7(8.5)	32(9.5)	
PAU	2(2.6)	5(5.7)	0(0)	0(0)	7(2.1)	
Presence pain at admission, n (%)	69(89.6)	76(87.4)	86(93.5)	72(87.8)	303(89.6)	0.524
Refractory pain, n (%)	2(2.6)	1(1.1)	7(7.6)	5(6.1)	15(4.4)	0.137
Refractory hypertension, n (%)	0(0)	0(0)	1(1.1)	1(1.2)	2(0.6)	0.593
Rupture or impending rupture, n (%)	1(1.3)	4(4.6)	4(4.3)	3(3.7)	12(3.6)	0.662
Operative procedure, n (%)						0.246
TEVAR	64(83.1)	77(88.5)	80(87.0)	64(78.0)	285(84.3)	
Hybrid operation	13(16.9)	10(11.5)	12(13.0)	18(22.0)	53(15.7)	
No. of stent, n (%)						0.573
1	67(87.0)	78(89.7)	85(92.4)	76(92.7)	306(90.5)	
≥2	10(13.0)	9(10.3)	7(7.6)	6(7.3)	32(9.5)	
Transfusion, n (%)	0(0)	0(0)	2(2.2)	4(4.9)	6(1.8)	0.056

AD, aortic dissection; IMH, intramural hematoma; AA, aortic aneurysm; PAU, penetrating aortic ulcer; TEVAR, thoracic endovascular aortic repair; FT4, free thyroxine.

### Univariate analysis and multiple logistic regression analysis at early follow-up

All 338 patients completed 30 days of follow-up, and the incidence of patients with ARAR was 4.1%. The univariate analysis indicated that there were 3 variables (other than thyroid hormones) associated with early ARAR, including glomerular filtration rate (GFR), cerebrovascular diseases (CVD), and operative procedure. Multiple logistic regression showed that, FT4 levels (OR 0.079, 95% CI 0.004 to 1.074, *P* = 0.105) were not associated with 30-day ARAR when FT4 was analyzed as a continuous variable. When all participants were stratified into quartiles based on FT4 levels, the lowest FT4 quartile group were at significantly greater risk of 30-day ARAR (OR 10.105, 95% CI 1.103 to 92.615, *P* = 0.041) compared to the highest FT4 quartile group. There was no significant association between 30-day ARAR and neither FT3 (OR 3.288, 95% CI 0.780 to 13.859, *P* = 0.105) nor TSH OR 0.508, 95% CI 0.242 to 1.064, *P* = 0.073). Besides FT4, GFR (OR 0.972, 95% CI 0.946 to 0.999, *P* = 0.044) was another multivariable predictor of ARAR. The results of multivariable logistic analyses for ARAR within 30 days are shown in Table 3.

**Table 3:** Multiple logistic regression analysis for 30-day aorta-related readmissions.

	OR	95% CI	*P value*
FT3	3.288	0.780–13.859	0.105
TSH	0.508	0.242–1.064	0.073
FT4 groups			
Q4	Reference		
Q1	10.105	1.103–92.615	0.041
Q2	3.008	0.292–30.939	0.354
Q3	3.871	0.403–37.201	0.241
GFR	0.972	0.946–0.999	0.044
CVD	2.671	0.835–8.547	0.098
Operative Procedure	2.055	0.558–7.566	0.279

FT3, free triiodothyronine; TSH, thyroid-stimulating hormone; FT4, free thyroxine; GFR, glomerular filtration rate; CVD, cerebrovascular diseases; Q1, quartile 1; Q2, quartile 2; Q3, quartile 3; Q4, quartile 4; OR, odds ratio; CI, confidential interval.

The incidence of patients with early ARAE was 2.7%. Operative procedure and blood transfused were variables significantly associated with ARAE on univariate analysis. The results of multiple logistic regression analysis showed that FT4 (OR 0.543, 95% CI 0.022 to 13.697, *P* = 0.711) was not associated with 30-day ARAE when FT4 was analyzed as a continuous variable. When all participants were stratified by quartiles of FT4, no significant associations were noted between 30-day ARAE and the lowest FT4 quartile group (OR 2.097, 95% CI 0.228 to 19.307, *P* = 0.513), FT3 (OR 3.168, 95% CI 0.688 to 14.587, *P* = 0.139), and TSH (OR 1.016, 95% CI 0.692 to 1.491, *P* = 0.937).

### Univariate analysis and multiple logistic regression analysis at mid-term follow up

A total of 288 patients (85.2%) were followed-up with regard to 12-month ARAR; the percentage of patients that encountered ARAR was 13.5%. Potential risk factors for mid-term ARAR identified by univariate analysis were gender, tobacco abuse, peripheral arterial disease (PAD), CVD, GFR, and stage of aortic diseases. After adjustment for these factors, FT4 (OR 0.114, 95% CI 0.018 to 0.705, *P* = 0.019) was noted to be significantly associated with 12-month ARAR when FT4 was analyzed as a continuous variable. When all participants were stratified by quartiles of FT4, the risk of ARAR in subjects at the lowest FT4 quartile (OR 5.687, 95% CI 1.708 to 18.935, *P* = 0.005) and the 2nd quartile (OR 3.635, 95% CI 1.102 to 11.990, *P* = 0.034) was significantly greater than those subjects at the highest quartile. FT3 and TSH were not significantly associated with 12-month ARAR (OR 0.563, 95% CI 0.231 to 1.369, *P* = 0.205; OR 0.914, 95% CI 0.744 to 1.123, *P* = 0.393). Other multivariable predictors of ARAR were CVD (OR 3.161, 95% CI 1.392 to 7.177, *P* = 0.006) and chronicity (OR 3.177, 95% CI 1.247 to 7.790, *P* = 0.015) (Table 4).

**Table 4:** Multiple logistic regression analysis for 12-month aorta-related readmissions.

	OR	95% CI	*P value*
Gender	0.299	0.067–1.323	0.112
Tobacco abuse			
None	Reference		
Current	1.293	0.476–3.512	0.614
Former	1.466	0.428–5.013	0.542
PAD	0.983	0.447–2.163	0.967
CVD	3.161	1.392–7.177	0.006
GFR	0.987	0.97–1.005	0.160
Stage of aortic diseases			
Acute	Reference		
Sub-acute	1.967	0.775–4.997	0.155
Chronic	3.117	1.247–7.790	0.015
FT3	0.563	0.231–1.369	0.205
TSH	0.914	0.744–1.123	0.393
FT4 groups			
Q4	Reference		
Q1	5.687	1.708–18.935	0.005
Q2	3.635	1.102–11.990	0.034
Q3	2.396	0.711–8.070	0.159

PAD, peripheral arterial disease; CVD, cerebrovascular diseases; GFR, glomerular filtration rate; FT3, free triiodothyronine; TSH, thyroid-stimulating hormone; FT4, free thyroxine; Q1, quartile 1; Q2, quartile 2; Q3, quartile 3; Q4, quartile 4; OR, odds ratio; CI, confidential interval.

Meanwhile, a total of 292 patients (86.4%) were followed-up with regard to 12-month ARAE; the incidence of 12-month ARAE was 8.9%. The results of univariate analysis showed that variables that were significantly associated with mid-term ARAE included gender, hypertension, and transfusion. Multiple logistic analysis revealed that FT4 (OR 1.015, 95% CI 0.640 to 1.608, *P* = 0.950) was not significantly associated with 12-month ARAE when FT4 was analyzed as a continuous variable. When all participants were stratified by quartiles of FT4, no significant associations were noted between 12-month ARAE and the lowest FT4 quartile group (OR 0.695, 95% CI 0.207 to 2.332, *P* = 0.556), FT3 (OR 1.108, 95% CI 0.449 to 2.733, *P* = 0.824), and TSH (OR 0.998, 95% CI 0.803 to 1.239, *P* = 0.983).

## Discussion

This study showed that among subjects who underwent TEVAR, those who had low-normal levels of FT4 were at increased risk of ARAR compared to those with high-normal levels of FT4 in both early and mid-term follow-up.

The entire circulatory system and thyroid are closely linked by their embryological anlage. The metabolic functions and the effects of thyroid hormones on the heart and vasculature have been discussed in several studies. Thyroid hormones function in the maintenance of normal vascular remodeling. When the secretion of thyroid hormones is disordered, the altered blood coagulability and endothelial dysfunction will affect the remodeling of the damaged aorta, resulting in increased risk in developing aorta-related clinical symptoms and possible readmission^15^.

In spite of this, the relationship between subclinical hypothyroidism and cardiovascular diseases has not been fully elucidated. One of the most common cardiovascular manifestations of hypothyroidism is hypertension, which results from increasing systemic vascular resistance. Hypertension can be aggravated by hypothyroidism^4^. At the same time, hypertension can also lead to poor prognosis for patients after TEVAR^10,16^. Therefore, lower levels of thyroid hormones may cause patients to be at a higher risk of readmission.

Lower levels of FT4, even within the normal range, can influence increased arterial stiffness, coronary artery calcification, atherosclerosis, cardiac pump performance, and C-reactive protein^4,17-20^. These clinical characteristics are also predictive factors of the adverse outcomes in patients after TEVAR^21–23^. Owing to the aforementioned effects of thyroid hormones on the overall vascular system, it is reasonable to find an association between low-normal levels of FT4 and increased risk of ARAR in patients after TEVAR.

In addition to reflection of the negative feedback of T4 and T3, TSH also embodies other influences, such as drugs and non-thyroidal disease^24^. FT3 and FT4 levels are differentially associated with some cardiovascular risk markers in euthyroid subjects^25^. Therefore, it is plausible that the levels of FT4 reflect a more sensitive index of cardiac “thyroid status”. This could explain why in this study, FT4, but not TSH and FT3, was an independent predictor of aortic readmission. Similar findings on the relationship of thyroid hormones and cardiovascular diseases were also reported in previous studies^9,25^.

Monitoring both ARAE and ARAR could provide additional information than monitoring only one of these parameters. Some researchers have found an inverse relationship between the risk of adverse events and the risk of readmission. In other words, patients who are at higher risk of readmission are at lower risk of adverse events or mortality^26–28^. For patients after TEVAR, several studies focused on adverse events and ignored readmissions. However, the risk of readmission partially embodies the quality of health and hospital services. Therefore, those two endpoints were collected to obtain more information about the prognosis of patients after TEVAR. In this study, FT4 was associated with ARAR instead of adverse events. These findings are also consistent with studies on other predictors of adverse events and readmission. Factors that are weak predictors of readmission tend to be strong predictors of adverse events, and vice versa^29,30^. As a rationale for our study, there might be no relationships between readmissions and adverse events. The detailed relationship should be assessed in future studies.

There are several limitations in this study. Firstly, it was a retrospective, single-center study. Some variables that affect thyroid hormones were also not considered in our study. In subsequent researches, we will consider more variables that affect thyroid hormones. Secondly, the predictive value of overt thyroid disorders in patients after TEVAR was not analyzed because the number of patients with overt thyroid disorders was too small for the analysis to be effective. This should be assessed in future studies as well.

## Conclusions

This study showed that, in comparison to patients with high-normal FT4 levels, patients with low-normal FT4 levels had greater risk of experiencing ARAR after TEVAR during the early and mid-term follow-up periods. In contrast, risk of ARAE was not significantly different in patients with differing FT4 levels. These findings need to be further verified in larger clinical studies before they can provide remarkable contributions to the prognostic assessment of patients after TEVAR.
